# A systems biology approach uncovers a gene co-expression network associated with cell wall degradability in maize

**DOI:** 10.1371/journal.pone.0227011

**Published:** 2019-12-31

**Authors:** Clément Cuello, Aurélie Baldy, Véronique Brunaud, Johann Joets, Etienne Delannoy, Marie-Pierre Jacquemot, Lucy Botran, Yves Griveau, Cécile Guichard, Ludivine Soubigou-Taconnat, Marie-Laure Martin-Magniette, Philippe Leroy, Valérie Méchin, Matthieu Reymond, Sylvie Coursol

**Affiliations:** 1 Institut Jean-Pierre Bourgin, INRA, AgroParisTech, CNRS, Université Paris-Saclay, Versailles, France; 2 Institute of Plant Sciences Paris-Saclay, CNRS, INRA, Université Paris-Sud, Université Evry, Université Paris-Saclay, Gif-sur-Yvette, France; 3 Institute of Plant Sciences Paris-Saclay, CNRS, INRA, Université Paris-Diderot, Sorbonne Paris-Cité, Gif-sur-Yvette, France; 4 Génétique Quantitative et Evolution—Le Moulon, INRA, Université Paris-Sud, CNRS, AgroParisTech, Université Paris-Saclay, Gif-Sur-Yvette, France; 5 UMR MIA-Paris, AgroParisTech, INRA, Université Paris-Saclay, Paris, France; 6 GDEC, INRA, UCA, Clermont-Ferrand, France; University of Sao Paulo, BRAZIL

## Abstract

Understanding the mechanisms triggering variation of cell wall degradability is a prerequisite to improving the energy value of lignocellulosic biomass for animal feed or biorefinery. Here, we implemented a multiscale systems approach to shed light on the genetic basis of cell wall degradability in maize. We demonstrated that allele replacement in two pairs of near-isogenic lines at a region encompassing a major quantitative trait locus (QTL) for cell wall degradability led to phenotypic variation of a similar magnitude and sign to that expected from a QTL analysis of cell wall degradability in the F271 × F288 recombinant inbred line progeny. Using DNA sequences within the QTL interval of both F271 and F288 inbred lines and Illumina RNA sequencing datasets from internodes of the selected near-isogenic lines, we annotated the genes present in the QTL interval and provided evidence that allelic variation at the introgressed QTL region gives rise to coordinated changes in gene expression. The identification of a gene co-expression network associated with cell wall-related trait variation revealed that the favorable F288 alleles exploit biological processes related to oxidation-reduction, regulation of hydrogen peroxide metabolism, protein folding and hormone responses. Nested in modules of co-expressed genes, potential new cell-wall regulators were identified, including two transcription factors of the group VII ethylene response factor family, that could be exploited to fine-tune cell wall degradability. Overall, these findings provide new insights into the regulatory mechanisms by which a major locus influences cell wall degradability, paving the way for its map-based cloning in maize.

## Introduction

Maize (*Zea mays*) is one of the most important staple crops, providing carbohydrates, proteins, lipids and vitamins for billions of people. It also serves as an important energy resource for ruminant animal. Indeed, maize silage is largely used for cattle feeding during winter and summer seasons, as well as a complementary resource with high-energy content in cow diets during the year [1]. Thus, improved feeding value is a key target of silage maize breeding.

Investigations with sheep in digestibility crates have shown that silage maize energy value is first related to cell wall degradability [2], which refers to the complex characteristics of cell wall to protect its carbohydrates from degradation by enzymes [3–6]. Importantly, *in vivo* cell wall degradability ranges from 36 to 60% in silage maize [2]. Correlatively, a similar two-fold variation was observed for *in vitro* cell wall degradability, which varies from 25 to 50% when determined as neutral detergent fiber (NDF) digestibility (IVNDFD) [7,8]. Consequently, numerous studies have been successfully carried out in silage maize to identify genomic regions (quantitative trait loci; QTL) involved in the variation of cell wall degradability [9–25]. Overall, they demonstrated that cell wall degradability is a highly multifactorial trait, primarily related to lignin content, lignin structure, hemicellulose content, and *p*-hydroxycinnamic acid cross-linkages [7,23,26–28].

In the majority of cases, QTL with ‘minor’ R^2^ values (R^2^ up to 15%) have been identified for cell wall component and degradability traits [23,29]. However, in the QTL studies of the maize recombinant inbred line (RIL) population derived from the cross between early dent lines F288 and F271, a cluster of 10 stable QTL with ‘major’ effects (R^2^ values up to 42.5% for cell wall components and accounting for 33.1% of the observed variation for IVNDFD) was mapped in bin 6.05 of the maize chromosome 6 [22]. The identified QTL colocalized in the 124–134 cM interval (referred to as QTL6.05), corresponding to the physical position 150.4–153.8 Mp in the B73 reference genome (AGPv3 sequence release). It is noteworthy that cell wall degradability of F271 was reported to be lower than that of F288 and, according to QTL detection [22], alleles of F288 at QTL6.05 increased cell wall degradability in the RIL population derived from F288 x F271.

The dissection of highly complex traits into their underlying components offers practical, technical, and commercial advantages. Specifically, trait dissection leads to the identification of phenotypic attributes which can be measured with greater precision and at a lower cost, but most importantly, these component traits typically display simpler inheritance patterns and provide additional information as to how genes and their variants act together in biological pathways to influence trait variation [30]. However, in complex genomes such as maize which exhibits extensive genome content variation among lines [31–37], including massive expansion of gene families, fine mapping remains a non-trivial process. Despite several attempts [29,38], the mechanisms and genes underlying the QTL6.05 effects remain largely unresolved. Selection of near-isogenic lines (NILs) have been performed to validate and fine map QTL in several plant species including maize [39]. Additionally, coupling genetic information with molecular phenotypes (e.g. transcript levels) can contribute to a better mechanistic understanding of trait variation [35,40–43].

Here, we implemented a multiscale systems approach to shed light on the mechanisms by which the QTL6.05 locus influences cell wall degradability. We developed and selected NILs in which the favorable F288 alleles at an 18–20 Mb region encompassing the QTL6.05 (referred to as the introgressed QTL6.05 region or QTL6.05^i^) had been introgressed into the background of the recurrent parent F271. We demonstrated that substitution of F271 alleles with that of F288 at QTL6.05^i^ led to variation in cell wall degradability. Using Illumina RNA sequencing (RNA-seq) datasets from NIL internodes, we then captured the QTL6.05^i^ allelic variation consequences at the transcript level relative to a maize reference genome that included F271- and F288-specific genomic sequences at the targeted QTL6.05 locus. Finally, we explored how the variation in gene expression contribute to the variation in cell wall-related traits observed between the selected NILs. Genes associated with the cell wall-related traits were identified, paving the way for pinpointing the causal genes at the QTL6.05 locus in future follow-up studies.

## Materials and methods

### Plant materials

The RIL122 from the F288 x F271 RIL progeny was crossed with F271 to produce F1 seeds. Then, 31 BC1 lines from the F1 plants x F271 progeny were backcrossed with F271. Genotyping of 31 BC2 lines (25 plants sown per BC1 progeny) was performed using a 50K single-nucleotide polymorphism chip from TraitGenetics. After quality controls based on missing data, heterozygosity, and minor allele frequency, 12,758 markers were retained for genotyping and two BC2 lines (30–16 and 31–04) were selected (S1 Table). Genotyping of BC2S1 lines (30–16 progeny referred to as 1^F271^ and 1^F288^; 31–04 progeny referred to as 2^F271^ and 2^F288^) was confirmed by High Resolution Melting (S2 Table). The selected BC2S2 lines were then grown in the field at Mauguio (Hérault, France) in 2015 with two row replicates. Irrigation was applied during summer to prevent water stress. For RNA-seq analysis, the bottom third of upstream-ear internodes (without nodes) of three representative plants in each of the two replicates were harvested two days before silking at tasseling stage. All samples were immediately frozen in liquid nitrogen and stored at -80°C. Because of the presence of borer on the stem in two plants, one of the two 1^F288^ samples was removed from the downstream analysis. For cell wall investigations, ears were removed by hand from plants at silage stage the day before and/or the day of harvest. Representative samples of 1 kg chopped material per plot were collected, dried in a ventilated oven 72 h at 55°C and ground with a hammer mill gondard to pass through a 1-mm screen. Genotyping of BC2S2 lines was further assessed by PCR (S2 Table and S1 Fig).

### Cell wall composition and degradability assessment

Dried samples from plants without ears were dried at 50°C for at least 14 h to exclude any moisture, before scanning through the Antaris II near-infrared reflectance analyzer (ThermoFisher). Briefly, for each sample, the measurement consisted of an average of 16 scans done while rotating the cups over the 10,000 cm-1–4,000 cm-1 range with a resolution of 8 cm-1, resulting in absorbance data every 4 cm-1. The resulting spectra were loaded into the TQ analyst software (v.9.4.45; ThermoFisher) and smoothed with a running average in a window size of 9 data points. Their first derivate was computed, normalized and used to estimate the cell wall-related traits of each sample using a formula that was previously calibrated for plants without ears [16]. A two-way ANOVA analysis was then conducted using R software (v.3.4.1; http://cran.at.r-project.org) as described [44].

### Pangenome construction

Oriented reads (sizing 150) of both F288 and F271 1.5 Mb-genomic sequences which included the QTL6.05 peak were first simulated using wgsim (v.0.3.0, depth = 20). Mapping of these simulated reads and AGPv3.22 real reads to one parental DNA sequence was then performed using Bowtie2 (v2.2.3, sensitive parameter). Then 2,322 representative transcript assemblies (RTAs) [34] that were lower than 45% coverage and 80% identity to the AGPv3.22 or absent in the AGPv3.22 sequence were mapped to both genomic sequences using gmap (v.2011-08-15, default parameter). Genomic sequences aligned with at least one of the previous sequences were then hard masked with N using maskfasta (v.2.25.0) with default parameters (S2 Fig).

### RNA-seq datasets

The internodes were crushed in liquid nitrogen using an Ika Mill crusher (IKA, Staufen-Im-Breisgau, Germany) prior RNA extraction. Total RNA was isolated using the TRIzol Reagent (Invitrogen) and purified with the Qiagen RNeasy Plant Mini kit and RNase-free DNase set according to the suppliers’ instructions. Total RNA integrity was evaluated using the Agilent 2100 bioanalyzer according to the Agilent technologies (Waldbroon, Germany). Individual Illumina TruSeq stranded RNA-seq libraries were prepared for each of the genotypes according to the supplier’s instructions. Libraries were sequenced in four lanes on the Illumina HiSeq 2000 platform at the Centre National de Séquençage (CNS, Evry, France) to generate paired-end stranded reads (sizing 260). For each library, RNA-Seq pre-processing was performed including adapters removing and quality control assessment with fastx toolkit (http://hannonlab.cshl.edu/fastx_toolkit/). The fastq datasets were trimmed for Phred Quality Score (Qscore >20, read length >30 bases), and free of ribosome sequences with tool sortMeRNA [45]. All steps of the experiment, from growth conditions to bioinformatic analyses, were managed in CATdb database [46] according to the international standard MINSEQE ‘minimum information about a high-throughput sequencing experiment’.

RNA-seq reads (35 million to 55 million quality-filtered RNA-seq reads per RNA-seq library) were then mapped to panB using TopHat2 (v. 2.0.14) without gene annotation file with minimum intron length set to 5 bp and maximum intron length to 60 kb, with default settings for other parameters. Next, transcript prediction was performed using Cufflinks (v.2.2.1, default parameters). Using bedtools getfasta (v.2.25.0, default parameters), coding sequences were then extracted leading to the CuffpanB dataset. To quantify expression levels, RNA-seq reads were mapped to panB using TopHat2 (v. 2.0.14) with a gene annotation file and the parameters described above. Expression quantification was determined using HTseq-count (v.0.6.0) with stranded orientation and ‘union’ option. The RNA-Seq project was submitted into the international repository NCBI-Gene Expression Omnibus [47] project ID GSE115157.

### Gene annotation

Genes were predicted using an improved and maize dedicated version of the TriAnnot pipeline (v.5.2p04) [48,49] as described below.

#### Step 1: Similarity search against mitochondria, chloroplast, non-coding RNAs and transposable elements, and masking

Mitochondrial and chloroplast similarities were identified using BLAST [50] against EMBL databanks release 129; non-coding RNAs were identified using tRNAscan [51], RNAmmer [52] and Infernal (http://infernal.janelia.org - infernal-1.1) based on RFAM database (EMBL RFAMEcm_v11). Transposable elements were identified using the maize transposable element database (http://maizetedb.org/~maize/). They were then all masked using RepeatMasker (cross_match engine, cutoff 250; http://repeatmasker.org).

#### Step 2: Similarity search against transcripts and related proteomes

This was performed using BLAST on the transposable element-masked sequence. Spliced alignments of BLAST hits were then generated using EXONERATE [53]. The following datasets were used: the CuffpanB dataset; all available Zea ESTs and full-length cDNAs (EMBL release 129), non-redundant proteomes of barley (UniProt Release 2016_03 and High confidence proteins IBGSC 2012), Brachypodium (UniProt Release 2016_10 and phytozome v11), rice (UniProt Release 2016_10, IRGSP 2005 and phytozome v11), Sorghum (UniProt Release 2016_10 and phytozome v11), Triticeae (UniProt Release 2016_10 and phytozome v11) and maize (UniProt Release 2016_10).

#### Step 3: Gene modeling

Two ab initio gene finders trained with a monocot and maize-specific matrix were used: FGeneSH (SOFTBERRY, http://linux1.softberry.com/berry.phtml) and AUGUSTUS [54] respectively. Evidence-driven gene prediction was also computed through the use of two different modules implemented in TriAnnot. The first module is based on BLASTX-EXONERATE spliced alignments of protein sequences from rice (phytozome v11), Brachypodium (phytozome v11), Sorghum (phytozome v11) and barley (High confidence proteins IBGSC 2012) which have been checked for Methionine start. In case of missing start and/or stop codons in the derived coding sequence model, iterative extension was applied in a range of 200 codons until an in frame start and stop codon could be found. If no start and/or stop codon could be find, the model was flagged as pseudogene. The second module (named SIMsearch, derived from FPGP pipeline [55] focused on similarity with maize transcripts: full-length cDNAs (EMBL release 129), CuffpanB and coding sequence-derived gene models from rice (phytozome v11 and IRGSP 2005), Brachypodium (phytozome v11), Sorghum (phytozome v11) and barley (High confidence proteins IBGSC 2012). For every locus showing similarity with transcripts, SIMSearch predicts the coding sequence borders by considering similarity with known proteins from related Poaceae.

#### Step 4: Selection and functional annotation of the best gene model at every locus

TriAnnot delivered six outputs with gene models: two ab initio gene finders, BLASTX-EXONERATE, and three SIMsearch-derived models. Therefore, a final step was required to select the best model at each locus. This was made according to a scoring system that considers the metrics of the alignments of each gene model against known proteins (taking into account the percentage of identity and coverage). Finally, if the retained gene model had a canonical structure but shared similarity over less than 70% of the length of its best BLAST hit, it was classified as pseudogene. Putative function for the best gene model was then assigned via a combination of similarity search (BLASTP) against several protein databanks, including Arabidopsis, Brachypodium, Hordeum, Glycine, Oryza, Prunus, Populus, Saccharum, Sorghum, Setaria and Vitis families, as well as the Pfam [56,57] protein domain collection using HMMER 3.0 (http://hmmer.janelia.org/software). The alignment with the best hit was parsed in order to check for the presence of gaps (>9 amino acids). TriAnnot follows a nomenclature based on the guideline established in 2006 by the IWGSC annotation working group and provides gene ontology (GO) terms for each gene model and protein domain predictions based on InterProScan [58] search against Pfam, Prosite [59], and SMART [60]. Final GFF output files were post-treated to be used with the graphical editor GenomeView [61]. Several round of manual expertise, made by different curators, were intensively followed to obtain a final structural and functional annotation of F271 and F288 DNA sequences at the targeted QTL6.05 locus (S3 Table).

### Differential expression analysis

RNA-seq data were normalized using TMM implemented in the edgeR package (v 3.18.1) [62,63] in R. Gene-wise RNA-seq counts were then analyzed using a negative binomial generalized linear model with a stepwise procedure for increasing the power to detect QTL6.05^i^-specific differentially expressed (DE) genes between NILs^F288^ (i.e. both 1^F288^ and 2^F88^) and NILs^F271^ (i.e. both 1^F271^ and 2^F271^) as follows:

In step I, the differential expression was assessed for the two pairs of NILs using a negative binomial generalized linear model with allele and NIL main effects, and an allele × NIL interaction. A Benjamini-Hochberg adjusted *P*-value of 0.05 was used as the cut-off criterion to identify genes with significant allele × NIL interactions.

In step II, the differential expression was assessed for each pair of NILs (e.g. 1^F288^ vs 1^F271^) using a negative binomial generalized linear model with allele main effect for each gene. A Benjamini-Hochberg adjusted *P*-value of 0.05 was used as the cut-off criterion to identify genes with significant allele effect for both NILs 1 and 2.

In step III, to identify DE genes that were not a result of NIL effects but were due to the QTL6.05^i^ region only, step II DE genes were further dissected by making the intersection between the step I genes with no significant differential expression for NIL effects (i.e. no significant differential expression for both NIL and allele × NIL interaction) and the step II DE genes for both NILs 1 and 2.

In the final step, the intersection between the step III DE genes for NILs 1 and the step III DE genes for NILs 2 delivered DE genes for which the differential expression between the NILs^F288^ and the NILs^F271^ was declared QTL6.05^i^-specific.

### Hierarchical clustering of gene expression profile

Hierarchical clustering analyses of the set of common QTL6.05^i^ DE genes were performed using Euclidean distances and unweighted pair group averages as the aggregation method with the R function *hclust*.

### Functional annotation of common QTL6.05^i^ genes

MapMan [64] was first used to identify homologous genes in Arabidopsis. GO term enrichment analysis based on the GO classification was then performed by comparing the relative occurrence of a GO term into each DE list (downregulated or upregulated gene list) to its relative occurrence in the genome (reference list) by a hypergeometric test with the R function *phyper*. A Benjamini-Hochberg adjusted *P*-value of 0.05 was used as the cut-off criterion.

### Correlation analyses

For correlation analysis between gene expression levels and cell wall-related traits, Pearson correlation coefficients (PCCs) were calculated between each DE gene and each trait over the two pairs of NILs using the R *cor* function. The Student’s *t*-distribution was used to test the significance of the PCC as follows: t = r/√[(1-r^2^)/(n-2)] with a level of significance set at 0.05 and n = 4, absolute PCCs higher than 0.95 were significant. The expression pattern of the set of DE genes correlating positively or negatively with at least one cell wall-related trait in both pairs of NILs were visualized using the R *cim* function from MixOmics (v. 6.3.1) [65].

Gene co-expression network analysis was performed to group DE genes into connected components based on absolute PCCs higher than 0.995 (level of significance of 0.01) and visualized using Cytoscape (v. 3.3.0) [66]. A node corresponded to a DE gene; an edge was determined by the similarity between expression profiles of paired genes calculated with PCCs. Connected modules were identified using NetworkAnalyzer (v. 2.7) [67].

### Promoter motif analysis

The presence of conserved motifs in the 5’ regions of the co-expressed genes was determined using the preferentially located motif (PLM) method [68], with minor modifications. Briefly, the dataset was set up using 1,000 bases upstream the transcription start site and 5’ untranslated region (UTR) from the maize AGPv3 plus [69] and the final GFF of the 1.5 Mb-genomic sequences from F271 and F288. Promoters with a 5’ UTR smaller than 10 bases were excluded in order to avoid false promoter regions. The final promoter dataset consisted of 119 promoter sequences, which were divided into two regions. First, the [−1,000, −300] region was used to learn the distribution model using a simple linear regression and determine a 95% confidence interval. Second, non-evenly distributed motifs (i.e. those exhibiting a peak above the confidence interval) were searched within the [−300, UTR] region, leading to the identification of 33 PLMs among 419 motifs tested coming from PLACE [70] and AGRIS [71].

### Quantitative RT-PCR analysis of candidate gene expression

Primer pairs were designed from the F271 and F288 1.5 Mb-genomic sequences at the QTL6.05 interval (S2 Table) using Primer-BLAST [72]. Quantitative RT (qRT)-PCR reactions were performed using the Bio-Rad CFX384 touch (Bio-Rad, France) and the SYBR Premix Ex Taq (Tli RNAseH Plus) (Takara, Ozyme, France) following the supplier’s instructions. A dilution series of the pooled cDNAs and a melting analysis were used to validate the primer pairs. Each sample was performed in duplicate. Gene expression was normalized with the mean of the two reference genes *ZmGRP2* (*GRMZM2G080603*) and *ZmUBC30* (*GRMZM2G102471*) coding for Glycine-rich RNA-binding protein 2 and Ubiquitin-conjugating enzyme E2, respectively. Differential expression for each gene was tested with a two-way ANOVA as described [44].

## Results

### Allelic variation at QTL6.05^i^ leads to higher cell wall degradability

**Fig 1:**
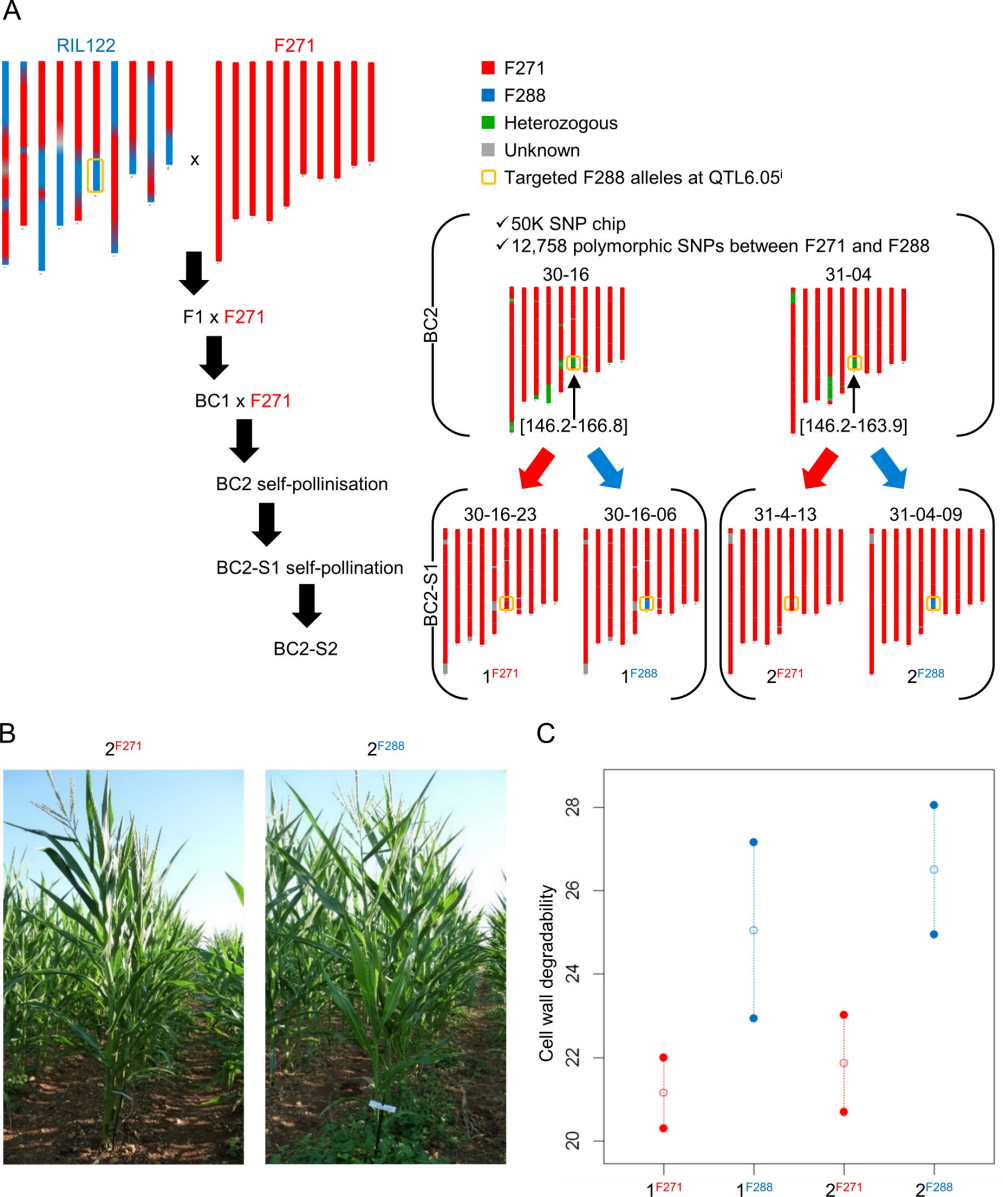
Selection and characterization of NILs with an introgressed genomic region at QTL positions for cell wall degradability. (A) Experimental workflow used for introgression of F288 alleles at an 18–20 Mb region encompassing the QTL6.05 (referred to as the introgressed QTL6.05 region or QTL6.05^i^) into the background of F271. SNP: single nucleotide polymorphism. (B) The selected BC2S2 NILs 1 (referred to as 1^F271^ and 1^F288^) and NILs 2 (referred to as 2^F271^ and 2^F288^) were grown in the field under well-watered conditions for DNA and mRNA sampling and phenotyping. (C) Comparison of the cell wall degradability IVNDFD performance of NILs introgressed with F288 alleles at QTL6.05^i^ (blue) and their respective recipient lines with F271 alleles (red). Open circles represent the means of two biological replicates.

**Fig 2:**
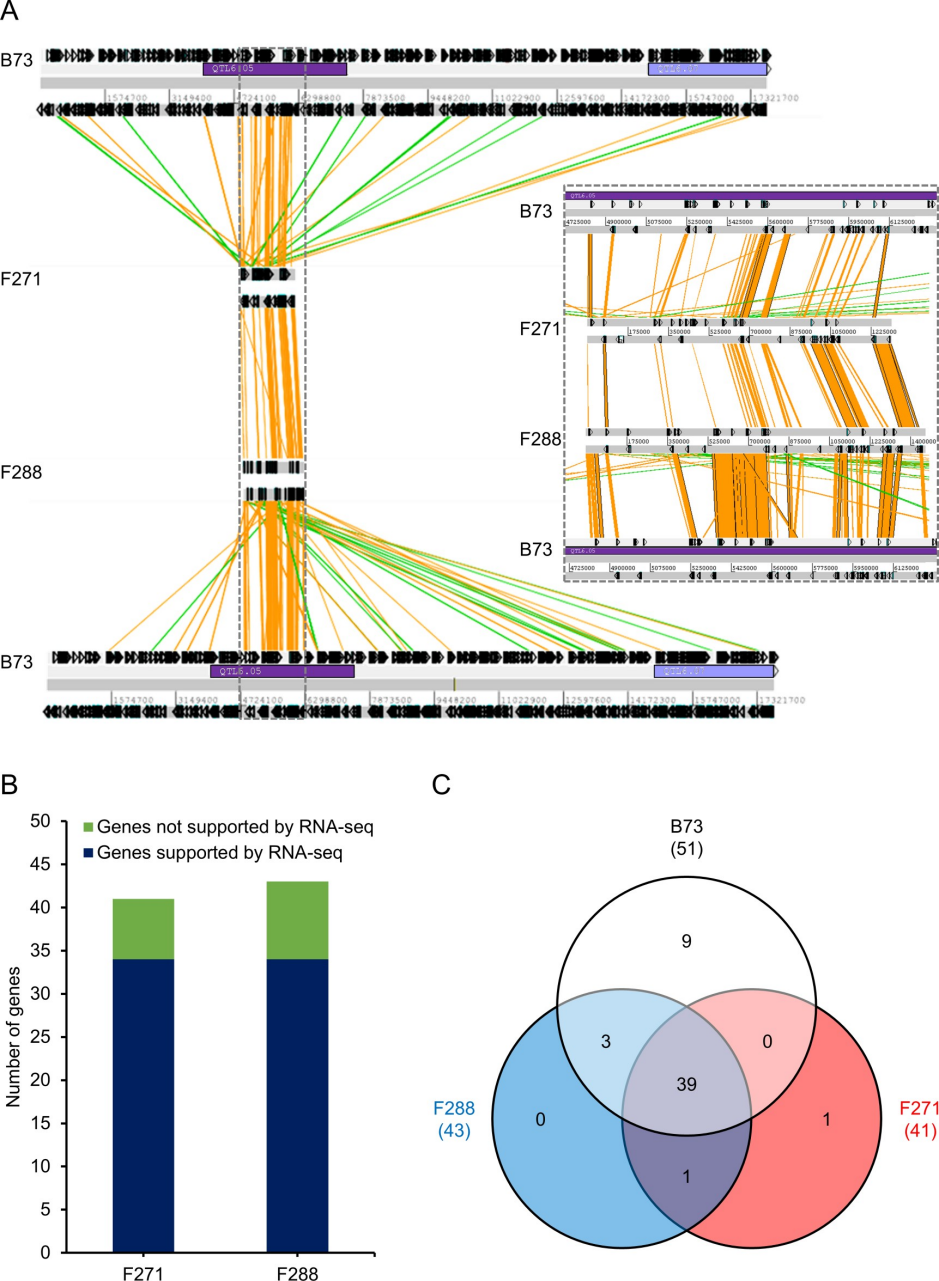
Structure of the introgressed genomic QTL6.05^i^ region. (A) Large structural variation between B73, F271 and F288 in the introgressed genomic QTL6.05^i^ region. Dark and light purple boxes represent clusters of QTL in bins 6.05 and 6.07, respectively, as previously defined [22]. The grey dotted line insertion represents a zoom of the genomic region that included the 1.5 Mb-genomic sequences from F271 and F288. Orange and green lines indicate sense and antisense DNA strands, respectively, of at least 910 bp and sharing at least 98% of identity; arrowed boxes represent genes and pseudogenes. (B) Number of annotated genes supported by RNA-seq predicted genes in the targeted QTL6.05 locus. (C) Gene content distribution in B73, F288 and F271 in the targeted QTL6.05 locus.

The RIL122 from the F288 x F271 RIL progeny carried F288 alleles at QTL6.05, although F271 alleles were already fixed at roughly more than half of the genome (Fig 1A) [21]. Therefore, this RIL was backcrossed with the parental inbred line F271 for three generations to fasten the introgression of F288 alleles at QTL6.05 into the background of F271. With the help of deep DNA genotyping, two BC2 lines (30–16 and 31–04) were selected that (1) showed a tremendous increase of F271 alleles fixed throughout the genome (93% and 94.3% for 30–16 and 31–04, respectively) compared to the RIL122 (53.8%) and (2) remained heterozygous at QTL6.05^i^ with a relatively similar introgressed genomic region size, ranging from 18 to 20 Mb (Fig 1A and S1 Table). According to QTL detection [22], the QTL6.05^i^ region included the QTL6.05 as well as a cluster of four QTL for cell wall component and degradability (referred to as QTL6.07) with ‘minor’ effects relative to QTL6.05 (R^2^ values ranging between 8 and 12% whatever the trait; Fig 2A). The two selected BC2 lines were then selfed twice to fix the introgressed genomic region at homozygous state and to multiply the progenies of 30–16 (1^F271^ and 1^F288^) and 31–04 (2^F271^ and 2^F288^) lines.

To assess the cell wall properties of the two pairs of NILs grown in the field (Fig 1B), near-infrared spectroscopy data were then collected as was done in the past for QTL6.05 detection [12,22]. The introgressed F288 alleles at QTL6.05^i^ conferred on the NILs 1^F288^ and 2^F288^ (NILs^F288^) a higher cell wall degradability (as estimated by IVNDFD) than the F271 alleles on the NILs 1^F271^ and 2^F271^ (NILs^F271^) (Fig 1B and 1C). Although the two pairs of NILs had similar lignin content, the NILs^F288^ exhibited lower cellulose content in the cell wall as compared to the NILs^F271^ (S4 Table). The NILs^F288^ also tended to have higher hemicellulose content in the cell wall as compared to the NILs^F271^ (S4 Table). Overall, these results evidenced that substitution of F271 alleles with those from F288 at the QTL6.05^i^ region led to cell wall composition variation and confirmed the presence of the detected QTL.

### Aligning RNA-seq reads to a pan-genomic reference genome: a tool to assess the number and type of genes predicted at the targeted QTL6.05 locus

RNA-seq datasets from internodes of the selected NILs were produced at tasseling stage in which transcript patterns of secondary cell wall cellulose synthase genes and lignin-related genes were shown to be highly abundant [73,74]. To take into account any genetic polymorphism, particularly putative present/absent genes, between F271 and F288 at the targeted QTL6.05 locus, a pan-genomic sequence (panB) was assembled through an *in lab* pipeline and used as a reference sequence for RNA-seq read mapping (Fig 2A and S2 Fig). This pan-genomic reference sequence included (1) the B73 transcript reference sequences (AGPv3), (2) 2,322 RTAs absent in B73 [34] and (3) genomic sequences specific to either F271 or F288 at the QTL6.05 locus which were kindly provided by the French Plant Genomic Resources Center (CNRGV) (Fig 2A and S2 Fig). The pipeline evidenced total lengths of 40.7 kb and 13.1 kb that were specific to F271 (3.1% of the F271 genomic region) and F288 (0.9% of the F288 genomic region), respectively.

To achieve better mapping of the RNA-seq reads to the panB reference sequence, gene prediction at the targeted QTL6.05 locus was then performed using (1) the TriAnnot pipeline [48] through *de novo* and similarity-based methods and (2) the transcript prediction based on the mapping of the RNA-seq dataset. About 80% of the predicted genes through the TriAnnot pipeline were supported by the mapped RNA-seq dataset (Fig 2B), highlighting the quality of the genomic sequences of each two lines at the QTL6.05 locus and the annotation. Of the 51 genes present in B73 at the targeted QTL6.05 locus, 39 (76%) were found in both F271 and F288 (Fig 2C and S3 Table). Three genes (genes *23*, *41* and *42*) were found only in F288 and B73; one gene (gene *43a*) provided by the RNA-seq dataset was found only in F288 and F271; one gene (gene *12*) was found only in F271. Overall, four genes (genes *12*, *23*, *41* and *42*) were present in only one of the two F271 and F288 parental inbred lines at the targeted QTL6.05 locus.

Mapping of the RNA-seq dataset to the panB reference sequence yielded 32,446 genes, including 1,198 RTAs. It is noteworthy that RNA-seq counts from the F271- and F288-specific genomic sequences present in the panB reference sequence were identified (including counts for genes *10*, *11*, *12*, *13* and *21*), highlighting the relevancy of the pan-genomic approach. Two (genes *23* and *41*) of the four genes present in only one of the two F271 and F288 parental inbred lines at the QTL6.05 locus did not exhibited measurable expression in all the experimental samples. In contrast, the gene *12* was expressed in all the experimental samples, although it was found only in F271 at the QTL6.05 locus (S3 Table). The gene *42* was consistently expressed at a very low level in the NILs 1^F288^ and 2^F288^, but not measurable in the NILs 1^F271^ and 2^F271^. Hence, this gene represents a case of single pattern expression (SPE) [75]. We designated an observed pattern as SPE_F288 if genes were expressed in the NILs^F288^ (i.e. in both 1^F288^ and 2^F288^), but not in the NILs^F271^ (i.e. in both 1^F271^ and 2^F271^). In contrast, we referred to genes that were expressed in the NILs^F271^, but not in the NILs^F288^ as SPE_F271.

### Allelic variation at QTL6.05^i^ leads to transcriptional variation

**Fig 3:**
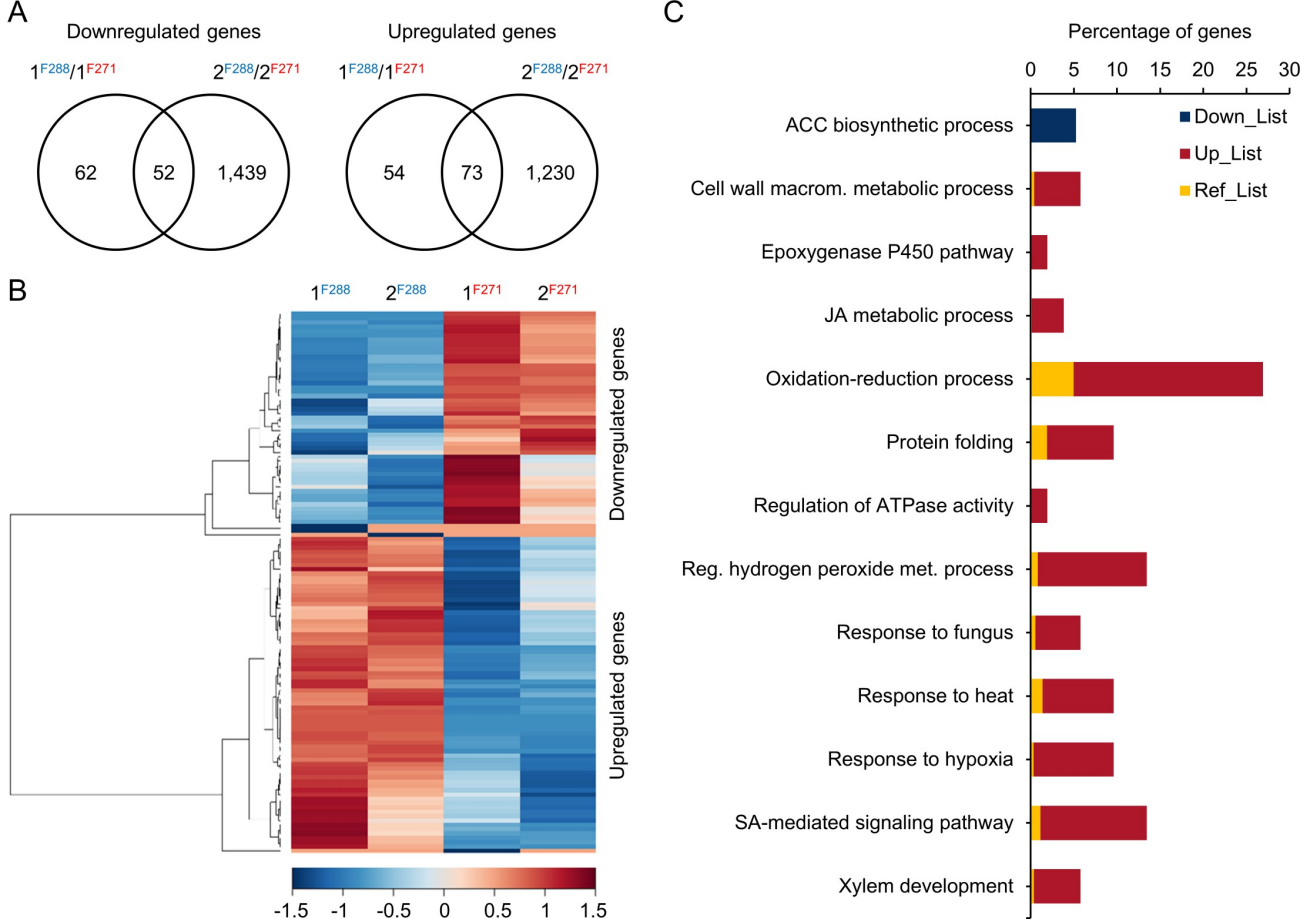
Transcriptomic adjustments in internodes of NILs introgressed at QTL6.05^i^. (A) Venn diagram of upregulated or downregulated genes in the NILs^F288^ relative to the NILs^F271^. (B) Hierarchical clustering of DE genes in the NILs^F288^ relative to the NILs^F271^. The indicated scale is the natural log value of the normalized level of gene expression. (C) GO Biological Process analysis of DE genes in the NILs^F288^ relative to the NILs^F271^. Only Biological Processes with a Benjamini and Hochberg adjusted *P*-value below 0.05 are represented. Down_List: analyzed downregulated gene list; Up_List: analyzed upregulated gene list; Ref_List: reference list; ACC biosynthetic process: 1-aminocyclopropane-1-carboxylate biosynthetic process; Cell wall macrom. metabolic process: Cell wall macromolecule metabolic process; JA metabolic process: jasmonic acid metabolic process; Reg. hydrogen peroxide met. Process: Regulation of hydrogen peroxide metabolic process; SA-mediated signaling pathway: salicylic acid-mediated signaling pathway.

To test the hypothesis that the substitution of F271 alleles with those of F288 at QTL6.05^i^ led to transcriptional variation, RNA-seq counts were analyzed using a stepwise procedure to detect DE genes upon the NILs^F288^ relative to the NILs^F271^ that depend only on the QTL6.05^i^ region. As shown in Fig 3A, this stepwise statistical analysis identified 241 and 2,794 DE genes for the NILs 1 and the NILs 2, respectively. Since our goal was to identify DE genes that were due to the introgressed QTL region, we ensured the robustness of the DE gene dataset by keeping only the DE genes that were common to both NILs. This selective approach delivered 125 DE genes, which are referred to as common QTL6.05^i^ DE genes (Fig 3A and S5 Table). As shown in Table 1, these included one RTA (cov45_id80_joint_Locus_58585), which highlighted once again the relevancy of the pan-genomic approach, and 18 genes located in QTL6.05^i^, of which five were located in the targeted QTL6.05 locus: (1) a *δ-1-pyrroline-5-carboxylate synthase* (gene *03*; *ZmP5CS1*), (2) an *aldo-keto reductase* (*AKR*) (gene *06a*; hereafter named *ZmAKR4C9* according to [76]), (3) a *FK506-binding protein* (gene *10*; hereafter named *ZmFKB20-1* according to [77]), (4) one gene (gene *14a*) with homology to Arabidopsis *ESMERALDA1 (ESMD1*), a putative *O*-fucosyltransferase [78], hereafter named *ZmESMD1*, and (5) a *40 S ribosomal protein S23* (gene *36*; *ZmRPS23*).

**Table 1:** Comprehensive list of the 125 common QTL6.05^i^ DE genes.

		Correlated cell wall-related traits					
AGPv3 gene ID and/or gene name	Trend in NIL^F288^	Cell wall degradabilty	Hemicellulose content	Cellulose content	Lignin content	Connected modules in the gene co-expression network	MapMan annotation
GRMZM2G051683	Down	-0.9968	-0.9825	0.9570	0.9668	M1	flavonoids.dihydroflavonols
**AC203754.4_FG008** ***ZmP5CS1* (gene 03)**	Down	-0.9955	-0.9853	0.9572	0.9769	M1	glutamate family.proline
GRMZM2G094666	Down	-0.9978	-0.9849	0.9597	0.9678	M1	myo-inositol.InsP-Kinases
GRMZM2G119766	Down	-0.9965	-0.9924	0.9693	0.9682	M1	not assigned
**GRMZM2G162127**	**Down**	-0.9825	-0.9810	0.9561	0.9633	M1	not assigned
Locus_58585^SPE_F271^	Down	-0.9918	-0.9978	0.9837	0.9469	M1	NA
GRMZM2G050917^SPE_F271^	Down	-0.9918	-0.9978	0.9837	0.9469	M1	not assigned
**GRMZM2G088349**	**Down**	-0.9724	-0.9936	0.9852	0.9264	M1	not assigned
GRMZM5G894619	Down	-0.9921	-0.9606	0.9187	0.9951	M1	ACC synthase*
GRMZM2G368838	Down	-0.9892	-0.9718	0.9374	0.9832	M1	AP2/EREBP, APETALA2
**GRMZM2G066153**	**Down**	-0.9673	-0.9606	0.9302	0.9612	M1	aromatic aa.tryptophan
GRMZM2G342509	Down	-0.9958	-0.9703	0.9326	0.9912	M1	cell wall proteins.LRR
GRMZM2G353276	Down	-0.9848	-0.9591	0.9188	0.9890	M1	cellulose synthesis.COBRA
**GRMZM2G106190**	**Down**	-0.9771	-0.9543	0.9146	0.9827	M1	ferredoxin
**GRMZM2G465835**	**Down**	-0.9986	-0.9743	0.9410	0.9821	M1	NAC domain transcription factor family
GRMZM2G467184	Down	-0.9954	-0.9693	0.9312	0.9917	M1	not assigned
GRMZM2G125649	Down	-0.9934	-0.9749	0.9478	0.9643	M1	not assigned
GRMZM2G463493	Down	-0.9916	-0.9614	0.9199	0.9944	M1	receptor kinases.leucine rich repeat XI
GRMZM2G170602	Down	-0.9850	-0.9396	0.8897	0.9996	M1	C1-metabolism
GRMZM2G040965	Down	-0.9735	-0.9235	0.8687	0.9990	M1	not assigned
GRMZM2G069335	Down	-0.9541	-0.9389	0.9016	0.9615	M1	not assigned
GRMZM2G430942	Down	-0.9874	-0.9485	0.9081	0.9794	M1	stress.biotic
GRMZM2G169382 *ZmERF71*	Up	0.9967	0.9937	-0.9720	-0.9657	M1	AP2/EREBP, APETALA2
GRMZM2G147399	Up	0.9986	0.9899	-0.9662	-0.9678	M1	development.unspecified
GRMZM2G108997	Up	0.9993	0.9890	-0.9637	-0.9720	M1	not assigned
GRMZM2G084794^SPE_F288^	Up	0.9866	0.9945	-0.9779	-0.9513	M1	protein.postranslational modification
**GRMZM2G140609** ***ZmRPS23* (gene 36)**	Up	0.9923	0.9977	-0.9816	-0.9529	M1	ribosomal protein.eukaryotic.40S subunit.S23
GRMZM2G085964 *ZmERF72*	Up	0.9817	0.9962	-0.9895	-0.9236	M1	AP2/EREBP, APETALA2
GRMZM2G142802	Up	0.9737	0.9982	-0.9968	-0.9102	M1	auxin.induced-regulated-responsive-activated*
GRMZM2G175805^SPE_F288^	Up	0.9918	0.9978	-0.9837	-0.9469	M1	development.late embryogenesis abundant
GRMZM2G098875	Up	0.9919	0.9941	-0.9790	-0.9465	M1	glutamate decarboxylase
**GRMZM2G059285** ***ZmAKR4C9* (gene 06a)**	Up	0.9856	0.9919	-0.9809	-0.9328	M1	minor CHO metabolism.others*
**GRMZM2G129234**	**Up**	0.9570	0.9914	-0.9923	-0.8969	M1	not assigned
**AC214451.3_FG005**^**SPE_F288**^	**Up**	0.9918	0.9978	-0.9837	-0.9469	M1	not assigned
GRMZM2G005344^SPE_F288^	Up	0.9918	0.9978	-0.9837	-0.9469	M1	not assigned
**GRMZM2G554314**^**SPE_F288**^	**Up**	0.9918	0.9978	-0.9837	-0.9469	M1	not assigned
AC203989.4_FG001	Up	0.9674	0.9781	-0.9601	-0.9405	M1	serine-glycine-cysteine group*
AC209215.4_FG004^SPE_F288^	Up	0.9827	1.0000	-0.9922	-0.9304	M1	transport.sugars
GRMZM2G136344	Up	0.9676	0.9957	-0.9932	-0.9111	M1	zeaxanthin epoxidase*
GRMZM5G859099	Up	0.9926	0.9643	-0.9307	-0.9740	M1	auxin.signal transduction
GRMZM2G131421	Up	0.9745	0.9603	-0.9256	-0.9732	M1	development.unspecified
GRMZM2G018820	Up	0.9975	0.9677	-0.9300	-0.9891	M1	glycerophosphodiester phosphodiesterase
GRMZM2G123480	Up	0.9875	0.9557	-0.9125	-0.9945	M1	not assigned
GRMZM2G050384	Up	0.9700	0.9598	-0.9274	-0.9665	M1	not assigned
GRMZM2G010673	Up	0.9994	0.9803	-0.9481	-0.9842	M1	not assigned
GRMZM2G387341	Up	0.9904	0.9671	-0.9292	-0.9889	M1	not assigned
GRMZM2G399136	Up	0.9924	0.9533	-0.9100	-0.9924	M1	stress.abiotic.heat
GRMZM2G009719	Up	0.9971	0.9667	-0.9277	-0.9919	M1	stress.abiotic.unspecified
**GRMZM2G026523**	**Up**	0.9830	0.9666	-0.9431	-0.9460	M1	transport.peptides and oligopeptides
GRMZM2G376661	Up	0.9847	0.9401	-0.8903	-0.9996	M1	allene oxidase synthase*
GRMZM2G004377	Up	0.9618	0.8989	-0.8373	-0.9969	M1	not assigned
GRMZM2G371462	Up	0.9628	0.9189	-0.8656	-0.9907	M1	not assigned
GRMZM2G012631	Up	0.9564	0.9184	-0.8676	-0.9828	M1	stress.abiotic.heat*
GRMZM2G069651	Up	0.9674	0.9159	-0.8594	-0.9971	M1	stress.abiotic.heat*
GRMZM2G316362	Up	0.9497	0.9391	-0.9044	-0.9539	M1	ACP desaturase*
GRMZM2G083716	Up	0.9364	0.9030	-0.8534	-0.9655	M1	protein.folding*
GRMZM2G024668	Up	0.9409	0.8949	-0.8384	-0.9783	M1	stress.abiotic.heat*
GRMZM2G028218	Up	0.9375	0.8816	-0.8196	-0.9823	M1	stress.abiotic.heat*
GRMZM2G164405	Down	-0.8584	-0.8310	0.7812	0.9004	M1	ACC synthase*
GRMZM2G092432	Down	-0.9418	-0.9350	0.9024	0.9445	M1	amino acid metabolism.misc
GRMZM2G302245	Up	0.8952	0.8675	-0.8190	-0.9298	M1	cell.division
GRMZM2G179685	Down	-0.8536	-0.8496	0.8145	0.8740	M1	flavonoids.dihydroflavonols
GRMZM5G881369	Down	-0.9043	-0.8614	0.8042	0.9496	M1	N-metabolism.misc
**GRMZM2G140763**	**Down**	-0.8631	-0.8682	0.8395	0.8721	M1	not assigned
GRMZM2G034623	Up	0.9224	0.9251	-0.8976	-0.9205	M1	not assigned
GRMZM2G177458	Up	0.9351	0.9353	-0.9069	-0.9323	M1	not assigned
GRMZM2G041714	Up	0.8949	0.8619	-0.8101	-0.9342	M1	not assigned
GRMZM2G113355	Up	0.9106	0.8732	-0.8196	-0.9500	M1	not assigned
GRMZM2G316721	Up	0.9060	0.9093	-0.8814	-0.9074	M1	not assigned
GRMZM2G440313	Up	0.8613	0.8396	-0.7934	-0.8975	M1	not assigned
GRMZM5G822947	Up	0.9161	0.8959	-0.8531	-0.9390	M1	not assigned
GRMZM2G056252	Up	0.8884	0.8765	-0.8377	-0.9097	M1	omega 6 desaturase*
GRMZM2G118610	Up	0.8853	0.9017	-0.8818	-0.8768	M1	phenylpropanoids.lignin biosynthesis.CAD*
GRMZM2G149422	Down	-0.8683	-0.8354	0.7826	0.9132	M1	signalling.in sugar and nutrient physiology
GRMZM2G128179	Down	-0.8939	-0.9152	0.8993	0.8775	M1	stress.abiotic.drought/salt
GRMZM2G300965	Up	0.8939	0.9187	-0.9053	-0.8733	M1	stress.biotic.respiratory burst*
GRMZM2G158328	Down	-0.9097	-0.8873	0.8427	0.9362	M1	WRKY domain transcription factor family
GRMZM2G173192	Up	0.9578	0.9018	-0.8537	-0.9596	M2	fermentation.LDH*
GRMZM2G035890	Up	0.9671	0.9278	-0.8913	-0.9491	M2	not assigned
GRMZM2G411216	Up	0.9528	0.8993	-0.8541	-0.9489	M2	not assigned
GRMZM2G165530	Down	-0.9452	-0.8774	0.8212	0.9613	M2	not assigned
GRMZM2G179827	Down	-0.9176	-0.9142	0.9069	0.8510	M2	ARR
GRMZM2G313272	Up	0.8990	0.8926	-0.8851	-0.8320	M2	aspartate family.asparagine
GRMZM2G124921	Up	0.9057	0.8377	-0.7870	-0.9095	M2	development.storage proteins
GRMZM2G126732	Up	0.8616	0.8439	-0.8321	-0.8003	M2	ethylene.synthesis-degradation*
GRMZM2G087186	Up	0.8691	0.8802	-0.8858	-0.7825	M2	fermentation.PDC*
GRMZM2G170958	Up	0.9004	0.9130	-0.9167	-0.8176	M2	HB,Homeobox transcription factor family
GRMZM2G178546	Down	-0.8441	-0.8457	0.8473	0.7627	M2	minor CHO metabolism.trehalose.TPP
GRMZM2G100158	Up	0.8619	0.8353	-0.8177	-0.8096	M2	misc.cytochrome P450*
GRMZM2G133407	Up	0.8471	0.8207	-0.8044	-0.7928	M2	misc.misc2
GRMZM2G021388	Up	0.9020	0.8502	-0.8120	-0.8838	M2	N misc.alkaloid-like
GRMZM2G008972	Down	-0.9132	-0.8350	0.7747	0.9349	M2	not assigned
GRMZM2G050556	Down	-0.9090	-0.8697	0.8399	0.8772	M2	not assigned
GRMZM2G552956	Down	-0.9096	-0.8857	0.8662	0.8612	M2	not assigned
GRMZM2G055802	Up	0.8067	0.8213	-0.8332	-0.7103	M2	not assigned
GRMZM2G119705	Up	0.8766	0.8460	-0.8247	-0.8304	M2	not assigned
GRMZM2G122543	Up	0.8544	0.8188	-0.7957	-0.8108	M2	not assigned
GRMZM2G127418	Up	0.8944	0.9171	-0.9271	-0.8029	M2	phenylpropanoids
GRMZM2G039993	Up	0.9138	0.8562	-0.8126	-0.9053	M2	salicylic acid.synthesis-degradation
GRMZM2G123973	Down	-0.9190	-0.8483	0.7937	0.9301	M2	transport.misc
GRMZM2G019183	Up	0.8756	0.8887	-0.8949	-0.7884	M2	trehalose.potential TPS/TPP
GRMZM2G101000	Up	0.7969	0.7826	-0.7393	-0.8373	M3	DNA.synthesis/chromatin structure
GRMZM2G311961	Down	-0.7418	-0.7117	0.6575	0.8051	M3	G-proteins
GRMZM2G381404	Down	-0.7918	-0.7719	0.7250	0.8383	M3	not assigned
GRMZM2G176774	Down	-0.7895	-0.7898	0.7559	0.8165	M3	protein.glycosylation
GRMZM2G015333	Down	-0.7922	-0.7849	0.7460	0.8265	M3	stress.biotic
**AC213621.5_FG002** ***ZmESMD1* (gene 14a)**	Up	0.9530	0.9601	-0.9552	-0.8857	M4	auxin.induced-regulated-responsive-activated
GRMZM2G023982	Up	0.9560	0.9694	-0.9676	-0.8852	M4	not assigned
**GRMZM2G035922** ***ZmFKBP20-1* (gene 10)**	Down	-0.9458	-0.9710	0.9766	0.8649	M4	protein.folding
**GRMZM5G869403** ***ZmEXO70B1***	**Up**	0.9289	0.9657	-0.9790	-0.8375	M4	RNA.regulation of transcription.unclassified
GRMZM2G464572	Down	-0.9551	-0.8847	0.8220	0.9870	M5	protein.degradation.ubiquitin.E2
GRMZM2G428391	Up	0.9290	0.8454	-0.7750	-0.9742	M5	stress.abiotic.heat*
GRMZM2G389416	Down	-0.6438	-0.5225	0.4477	0.6940	M6	glutamate family.proline
GRMZM2G086590	Down	-0.6438	-0.5225	0.4477	0.6940	M6	isoprenoids.terpenoids
GRMZM2G006206	Down	-0.9286	-0.9805	0.9947	0.8479	M7	G-proteins
GRMZM2G159393	Down	-0.9227	-0.9776	0.9960	0.8331	M7	misc.oxidases
**GRMZM2G077662**	**Down**	-0.9182	-0.9667	0.9744	0.8547	NA	G-proteins
**GRMZM2G067122**	**Up**	0.9408	0.9827	-0.9955	-0.8542	NA	not assigned
GRMZM2G431039	Down	-0.7376	-0.7720	0.7604	0.7348	NA	misc.beta 1,3 glucan hydrolases
GRMZM2G164074	Up	0.7721	0.7447	-0.7324	-0.7121	NA	misc.cytochrome P450*
GRMZM2G093246	Down	-0.5015	-0.6296	0.6882	0.3994	NA	misc.myrosinases-lectin-jacalin
GRMZM2G087824	Down	-0.8454	-0.7783	0.7336	0.8378	NA	not assigned
GRMZM2G071846	Up	0.8348	0.7815	-0.7156	-0.9027	NA	phosphoribosyltransferases.aprt
GRMZM2G054448^SPE_F288^	Up	0.6488	0.5684	-0.4839	-0.7639	NA	protein.degradation
GRMZM2G105348	Down	-0.6933	-0.6953	0.6608	0.7321	NA	stress.abiotic.heat

DE genes located at QTL6.05^i^ and/or displaying SPE patterns are in bold and/or underlined, respectively. Dark gray indicates DE genes with expression levels negatively and significantly correlating with cell wall-related traits. Light gray indicates DE genes with expression levels positively and significantly correlating with cell wall-related traits. Co-expression modules as determined by NetworkAnalyzer are designated M1-M7. Cell wall degradability, sugar content and lignin content were estimated by IVNDFD, hemicellulose content in % NDF, cellulose content in % NDF and acid detergent lignin in % NDF. Locus_58585: cov45_id80_joint_Locus_58585; NA: not appropriate.

* DE genes with significant enrichment in Biological processes.

This prompted us to compare our common QTL6.05^i^ DE gene dataset to the deregulated transcriptome of four RILs derived from the cross of F288 x F271 [38]. Interestingly, five of the 125 common QTL6.05^i^ DE genes exhibited DE patterns between F271 and the set of four RILs (Table 1): (1) *ZmFKB20-1* described above, (2) an *universal stress protein* (*USP*) (*GRMZM2G009719*), (3) the maize *J3* ortholog (*GRMZM2G028218*; *ZmHSP40*) encoding an HSP40 known to function in meristem size control in Arabidopsis [79], (4) one gene (*GRMZM2G041714*) with homology to Arabidopsis *hypoxia-induced gene domain 3*, and (5) *GRMZM2G169382*, an ortholog of Arabidopsis *group VII ethylene response factor (ERF-VII) ERF71/HYPOXIA RESPONSIVE ERF2* (*ZmERF71/HRE2*) that may supervise plant intracellular reactive oxygen species (ROS) homeostasis [80]. It is noteworthy that the gene *42* with unknown function did not belong to the common QTL6.05^i^ DE gene dataset, which is not in favor of a direct role of this gene in cell wall-related trait variation. Importantly, none of the common QTL6.05^i^ DE genes were located in the QTL6.07 confidence interval.

Of the common QTL6.05^i^ DE genes, 52 and 73 were found to be downregulated and upregulated in the NILs^F288^ relative to the NILs^F271^, respectively (Fig 3A and 3B, and Table 1). Interestingly, two of the 52 downregulated genes showed SPE_F271 patterns, while seven of the 73 upregulated genes showed SPE_F288 patterns (Table 1). Additionally, GO analysis revealed that the downregulated genes had significant enrichment in the “1-aminocyclopropane-1-carboxylate (ACC) biosynthetic process”, which is the first committed and in most instances the rate-limiting step in ethylene biosynthesis (Fig 3C and S5 Table). For the upregulated genes, enriched GO terms associated with biological processes were related to oxidation-reduction, regulation of hydrogen peroxide metabolism, response to hypoxia, jasmonic acid (JA) metabolism, salicylic acid (SA)-mediated signaling pathway, protein folding, response to heat, cell wall macromolecule metabolism and xylem development (Fig 3C and S5 Table).

### Almost two-thirds of the common QTL6.05^i^ DE genes are associated with the cell wall-related traits

To test the ability of the 125 common QTL6.05^i^ DE genes to be associated with the cell wall-related traits, linear correlation between gene expression levels and traits was determined by calculating PCCs between the expression level of each of the common QTL6.05^i^ DE genes and each cell wall-related trait. We found 79 DE genes significantly associated with at least one of the traits, referred to below as the correlating DE gene set (Table 1 and S6 Table). This included the cov45_id80_joint_Locus_58585 and the five DE genes (*ZmP5CS1*, *ZmAKR4C9*, *ZmFKB20-1*, *ZmESMD1* and *ZmRPS23*) located in the QTL6.05 locus (Table 1).

**Fig 4:**
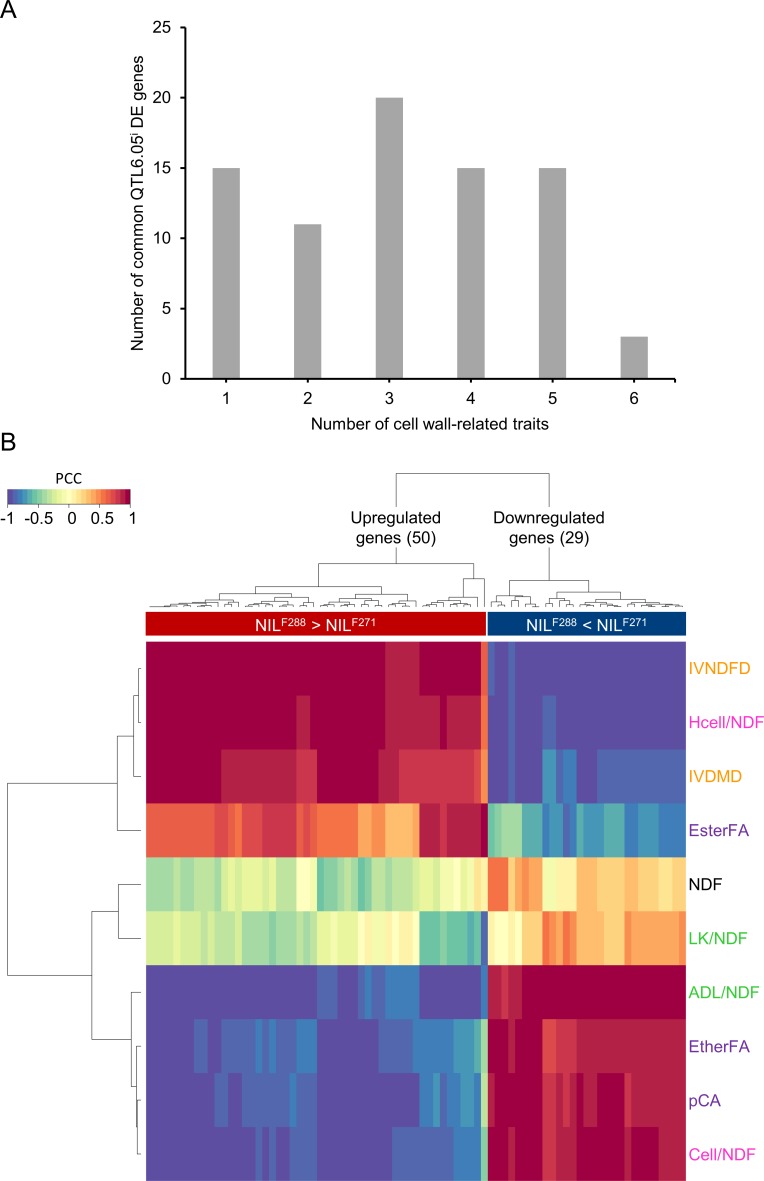
Identification of DE genes associated with cell wall-related traits. (A) Number of common QTL6.05^i^ DE genes correlating with at least one of the cell wall-related traits. (B) Gene expression levels of the 79 correlating DE genes in NILs introgressed into QTL6.05^i^. Columns represent gene expression profiles, and rows represent cell wall-related traits as follows: cell wall content (black), degradability (orange), sugar content (pink), lignin content (green) and *p*-hydroxycinnamic acids content (purple). IVNDFD: *in vitro* neutral detergent fiber digestibility; Hcell/NDF: hemicellulose content in % NDF; IVDMD: *in vitro* dry matter digestibility; EsterFA: esterified ferulic acid content in mg g^-1^ NDF; LK/NDF: lignin klason in % NDF; NDF: neutral detergent fiber; ADL/NDF: acid detergent lignin in % NDF; Cell/NDF: cellulose content in % NDF; pCA: *p*-coumaric acid content mg g^-1^ NDF; EtherFA: etherified ferulic acid content in mg g^-1^ NDF.

The number of correlating DE genes ranged from 15 for one trait up to 20 for three traits (Fig 4A and S6 Table). A subset of three upregulated genes (*AC209215*.*4_FG004*, *GRMZM2G085964* and *GRMZM2G142802)* were associated with six traits (S6 Table). Among them, one gene (*AC209215*.*4_FG004*) with homology to Arabidopsis *voltage dependent anion channel 1* showed SPE_F288 pattern (Table 1). Interestingly, the subset of three upregulated genes also included a transcription factor (TF) with homology with Arabidopsis *ERF72/RELATED TO APETALA2*.*3 (RAP2*.*3)* (*GRMZM2G085964*; hereafter named *ZmERF72/RAP2*.*3*) linked to the hypoxia response [81]. The third upregulated gene (*GRMZM2G142802*) was annotated as a stem-specific protein TSJT1 and related to enriched GO terms “SA-mediated signaling pathway”, “regulation of hydrogen peroxide metabolic process” and “hypoxia” (Table 1 and S5 Table).

The expression levels of the 79 correlating DE genes and their correlation with the cell wall-traits were visualized in a clustered map, revealing opposite correlation of individual DE genes with multiple traits (Fig 4B). It is worth noting that upregulated genes (including *ZmAKR4C9*, *ZmESMD1* and *ZmRPS23* located in the QTL6.05 locus) showed positive correlations with cell wall degradability and hemicellulose content, and negative correlations with cellulose content (Fig 4B). In contrast, downregulated genes (including *ZmP5CS1* and *ZmFKB20-1* located in the QTL6.05 locus) showed negative correlations with cell wall degradability and hemicellulose content, while exhibiting positive correlations with cellulose content (Fig 4B).

### Toward a co-expression network of genes associated with QTL6.05^i^

**Fig 5:**
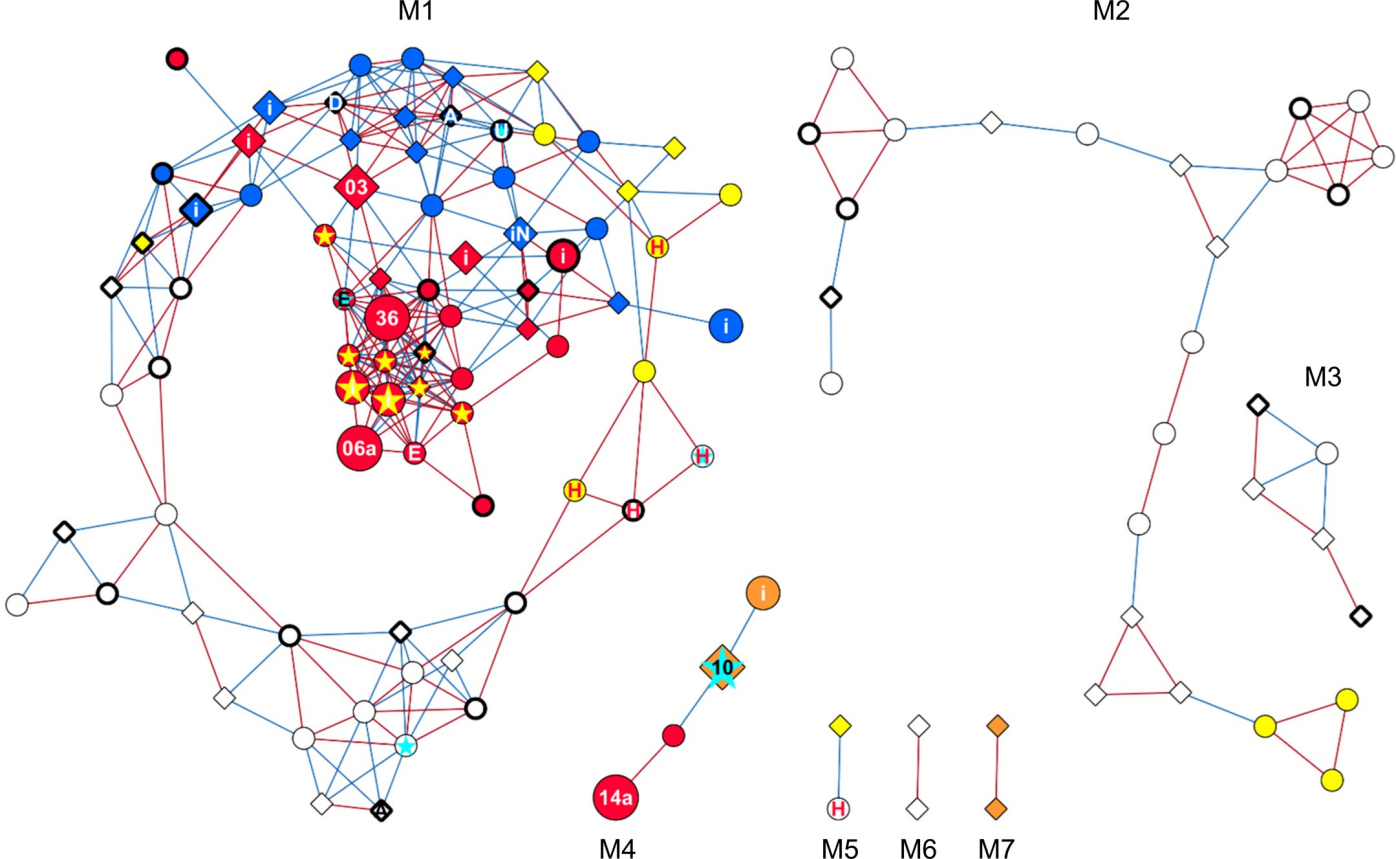
Co-expression network of common QTL6.05^i^ DE genes. Downregulated genes in the NILs^F288^ relative to the NILs^F271^ are represented by diamonds, and upregulated genes by circles. Colored diamonds indicate genes with expression levels negatively correlating with cell wall degradability (IVNDFD) and/or hemicellulose content (Hcell/NDF), and/or positively correlating with cellulose content (Cell/NDF). Colored circles indicate genes with expression levels positively correlating with IVNDFD and/or Hcell/NDF, and/or negatively correlating with Cell/NDF. Node colors represent correlation with IVNDFD (yellow), IVNDFD and Hcell/NDF (blue); IVNDFD, Hcell/NDF and Cell/NDF (red); Hcell/NDF and Cell/NDF (orange). The “i” present in the nodes indicates DE genes located in the QTL6.05^i^ region; numbered and larger nodes indicate genes located in the QTL6.05 locus (gene *03*: *ZmP5CS1*; gene *06*: *ZmAKR4C9*; gene *10*: *ZmFKB20-1*; gene *14a*: *ZmESMD1*; gene *36*: *ZmRPS23*). The letters “A”, “D”, “E”, “H”, “N” and “U” present in the nodes indicate genes encoding ACC synthase, DREB TF, ERF-VII TF, HSP, NAC TF and universal stress protein, respectively. Blue stars indicate DE genes previously identified [38]; yellow stars indicate DE genes with SPE patterns co-expressed with *ZmRPS23* (gene *36*). Thick borders indicate gene with the GCCGCC motif in their promoter. Edges colored blue connect co-expressed genes with a PCC less than -0.995, red edges represent a PCC bigger than 0.995. Modules as determined by NetworkAnalyzer are designated M1-M7.

To get more insights into the putative coregulation of the 125 common QTL6.05^i^ DE genes, a network analysis of co-expression relationships among the common QTL6.05^i^ DE genes was performed using PCCs between the gene expression levels. The resulting gene co-expression network incorporated 116 DE genes and 329 edges (Fig 5 and S7 Table). Seven connected modules (designated M1-M7) were determined and significant correlations with cell wall degradability and sugar content were visualized in the gene co-expression network for each DE gene (Table 1). As shown in Fig 5, modules M1 (77 DE genes and 283 edges), M4 (4 DE genes and 3 edges) and M7 (2 DE genes and 1 edge) brought together the majority of the correlating DE genes since 58% (45/77), 100% (4/4) and 100% (2/2) of their nodes, respectively, were correlated with at least two of the three cell wall-related traits. It is also worth noting that 70% (54/77) of the genes in module M1 correlated with cell wall degradability (Table 1). In contrast, DE genes within modules M2 (24 DE genes and 34 edges), M3 (5 DE genes and 6 edges), M5 (2 DE genes and 1 edge) and M6 (2 DE genes and 1 edge) tended to be almost not correlated with cell wall-related traits (Table 1 and Fig 5). Interestingly, up to two-thirds of the genes in module M1 that were associated with at least one of the three cell wall-related traits were related to the enriched biological processes we identified. One noteworthy gene of module M1 that negatively correlated with cell wall degradability and hemicellulose content was an *ACC synthase* (*ACS*) gene (*GRMZM5G894619*; hereafter named *ZmACS7*) involved in the ethylene biosynthetic pathway (Fig 5). Also related to hormone responses were the two *ERF-VIIs* (*ZmERF71/HRE2* and *ZmERF72/RAP2*.*3*) that showed positive correlation with the three cell wall-related traits (Fig 5). Another class of genes correlated with cell wall degradability are heat shock proteins (HSPs) known to affect protein folding and response to heat (Fig 5). Two positively correlating genes (*GRMZM2G012631* and *GRMZM2G069651*) with homology to Arabidopsis *HSP90* were connected to a limited set of other genes, including another maize *HSP90* ortholog (*GRMZM2G024668*) and *ZmHSP40*.

Of the 116 DE genes incorporated in the gene co-expression network, 16 (almost 14% of the DE genes incorporated in the gene co-expression network) located in QTL6.05^i^, including the fives genes (*ZmP5CS1*, *ZmAKR4C9*, *ZmFKB20-1*, *ZmESMD1* and *ZmRPS23*) located in the QTL6.05 locus (Table 1 and Fig 5). Furthermore, we observed that 13 of these 16 DE genes were in module M1 (including *ZmP5CS1*, *ZmAKR4C9* and *ZmRPS23*), while three were in module M4 (including *ZmFKB20-1* and *ZmESMD1*). All these 16 DE genes were correlating with at least two of the three cell wall-related traits. Of note, *ZmRPS23* in module M1 had high connectivity with 13 DE genes (including *ZmERF71/HRE2* and eight of the nine DE genes displaying SPE_patterns) that were all correlated with cell wall degradability and sugar content (Fig 5). Moreover, seven neighbors of *ZmRPS23* were connected with *ZmAKR4C9*, while four neighbors of *ZmRPS23* were connected with *ZmP5CS1*. It is also worth noting that *ZmAKR4C9* had high connectivity with eight DE genes (including *ZmERF2/RAP2*.*3*) that were all correlated with cell wall degradability and sugar content (Fig 5). Additionally, three of the four DE genes of module M4 were located in QTL6.05^i^, namely *ZmFKB20-1*, *ZmESMD1* and one gene (*GRMZM5G869403*) annotated as exocyst subunit EXO70 family protein B1 (*ZmEXO70B1*).

### Transcription factors associated with QTL6.05^i^

Genes having the same TF binding sites are assumed to be functional partners since they are regulated by the same TFs. Therefore, a PLM study [68] was carried out to determine whether there were any over-represented TF binding sites in the promoter of the common QTL6.05^i^ DE gene dataset. Among the promoters of the common QTL6.05^i^ DE genes, we identified the CATGTG motif (S8 Table) which is bound by NAC TFs [82]. Interestingly, there was one gene (*GRMZM2G465835*) in module M1 (Fig 5) located in QTL6.05^i^ and showing homology to Arabidopsis *NAC090*, which is a negative regulator of SA-mediated leaf senescence [83]. Also present in the promoters of the common QTL6.05^i^ DE genes were (1) the TGACG motif which is involved in transcriptional activation of several genes by auxin and/or SA [84] and (2) the GCCGCC motif which is the core site of GCC-box required for the binding of AP2/ERF TF family and for the regulation of JA-responsive gene expression [85,86]. It is noteworthy that in module M1 the two *ERF-VIIs* (*ZmERF71/HRE2* and *ZmERF72/RAP2*.*3*) were central in the sense that together they covered 73% (19/26) of the DE genes (including *ZmAKR4C9* and *ZmRPS23* located in the QTL6.05 locus) that were correlated with cell wall degradability and sugar content. Additionally, a member (*GRMZM2G368838*) of the DREB (dehydration-responsive-element-binding) subfamily A-4 of AP2/ERF TF family was also present in module M1 (Fig 5). One noteworthy neighbors of *GRMZM2G368838* was *ZmACS7* (Fig 5). Finally, several F271 or F288-specific PLMs were identified in the promoter of *ZmAKR4C9*, *ZmFKBP20-1*, *ZmESD1* and *ZmRPS23* (S3 Fig). Although, the biological importance of these genotype-specific motifs will require further investigation, these results, together with those of Fig 5, support *ZmAKR4C9*, *ZmFKBP20-1*, *ZmESD1* and *ZmRPS23* as candidate genes for the targeted QTL6.05 locus. Therefore, to confirm the differential expression, qRT-PCR experiments were performed based on the same samples that had been used for the initial RNA-seq analysis. Transcript levels of *ZmAKR4C9* were not quantified because it was not possible to design gene-specific primers of sufficient quality for qRT-PCR. The other three candidate genes were significantly affected by the substitution of F271 alleles with that of F288 at QTL6.05^i^ with opposite effects: (1) downregulation of *ZmFKBP20-1* in the NILs^F288^ relative to the NILs^F271^; (2) upregulation of *ZmESD1* and *ZmRPS23* in the NILs^F288^ relative to the NILs^F271^ (S9 Table). These results were in agreement with those obtained by RNA-seq (Table 1 and S5 Table), thus validating our differential expression analysis.

## Discussion

### Expanding maize genetic and genomic resources to shed light on the mechanisms whereby the QTL6.05 locus influences cell wall degradability

We implemented a systems biology approach that combined genetic, genomic, transcriptomic and phenotypic data to begin answering the mechanisms whereby the major QTL6.05 locus influences cell wall degradability. The use of the NILs that we developed using the F271 inbred line as the recurrent parent and the RIL122 carrying F288 alleles at QTL6.05 as donor enabled us to show that substitution of F271 alleles with those of F288 at QTL6.05^i^ led to an increase of four percentage points of IVNDFD, as previously reported for QTL6.05 in QTL mapping experiments [21,22]. Consequently, the results obtained with the selected NILs most likely reflected the QTL6.05 rather the QTL6.07, which had ‘minor’ effects [21,22]. Consistently, none of the 125 common QTL6.05^i^ DE genes were located in the QTL6.07 support interval. Additionally, similar to what we observed for cell wall degradability, we found that hemicellulose content tended to be higher in the NILs^F288^ compared to the NILs^F271^. In contrast, cellulose content in the NILs^F288^ was significantly lower relative to the NILs^F271^. These data are comparable to the previous characterizations of the F288 x F271 RIL progeny [12], as well as those of a forage maize doubled haploid population [23], an association panel [87] and six mapping populations derived from European elite maize germplasm [25]. Therefore, these results, together with those of cell wall degradability, showed that the NILs developed in this study provided the suitable biological tools to understand the underlying mechanisms and genes that control cell wall-related trait variation at QTL6.05.

Gene prediction at the QTL6.05 locus from both F271 and F288 inbred lines highlighted the three genes *23*, *41* and *42* that are present in F288 but not in F271, and the gene *12* that is present in F271 and not in F288. It is noteworthy that the gene *12* was expressed in the two pairs of NILs, which might reflect relocated single-copy gene in F288. In contrast, we evidenced 10 genes displaying SPE patterns, including the gene *42*. Hence, such SPE pattern was consistent with the absence of the gene *42* in F271 at the QTL6.05 locus interval and suggests that this gene is completely absent from the genome of F271. It was not possible to determine the fraction of genes displaying presence/absence variation among the nine other identified SPE patterns because of the lack of genomic data from both F271 and F288. Considering that low proportions of presence/absence variations had been observed among genes displaying SPE patterns [37,75,88,89], we anticipate that these nine genes (two-thirds of which have unknown function) represent cases of genotype-dependent differential gene expression. Alleles from the unfavorable NILs for cell wall degradability were activated in the favorable NILs for cell wall degradability presumably via interactions with QTL6.05^i^-specific regulatory factors from the favorable NILs. Consistently, the group of co-expressed genes that were located in QTL6.05^i^ included the maize *NAC090* ortholog involved in SA-mediated signaling pathway [83], which is one of the enriched biological processes observed here. Moreover, the nine SPE patterns were correlated with cell wall-related traits. This observation confirms the expectation that SPE can affect the phenotype variation directly [35,37] and suggests a biological role of these SPE patterns in the variation of cell wall degradability controlled by the QTL6.05 locus.

### Identification of hub genes associated with cell wall-related trait variation at QTL6.05^i^

Additionally, the systems biology approach employed in this study revealed biological processes occurring in response to allelic variation at QTL6.05^i^. First, a set of processes related to oxidation-reduction and regulation of hydrogen peroxide metabolism was included in the gene co-expression network. Two noteworthy genes are a *ferulate 5-hydroxylase* (*GRMZM2G100158*) and a *cinnamyl alcohol dehydrogenase* (*GRMZM2G118610*) involved in lignin biosynthesis [90]. In addition, two *plant cysteine oxidases* (*AC203989*.*4_FG001* and *GRMZM2G113355*; *PCOs*), one *ACC oxidase* (*GRMZM2G126732*) involved in the ethylene biosynthetic pathway, one *respiratory burst oxidase* homolog gene (*GRMZM2G300965* or *ZmRbohB*) encoding a NADPH-oxidase and *ZmAKR4C9* were also observed in the gene co-expression network. These results, together with those described previously [38], support the idea that a tight and complex regulation, a long way upstream of the monolignol biosynthetic pathway, is driven by the F288 alleles at QTL6.05^i^.

Second, genes involved in hormone responses are co-expressed in the network. Notably, *ZmACS7*, the maize PCO ortholog *AC203989*.*4_FG001* and the two *ERF-VIIs*, *ZmERF71/HRE2* and *ZmERF72/RAP2*.*3*, were co-expressed in module M1 and associated with cell wall-related trait variation. PCOs were shown to oxidize the penultimate cysteine of ERF-VIIs by using oxygen as co-substrate, thereby controlling the lifetime of these proteins in Arabidopsis [91,92]. A perspective is emerging in which a diversified set of mechanisms can influence *ERF-VII* expression in order to specify their functions in a wider network of physiological pathways, in particular those activated by hormones [81,93]. In that respect, a target of ERF-VIIs, namely hypoxia responsive universal stress protein 1, has been shown to coordinate oxygen sensing by PCO/ERF74 (also known as RAP2.12) with H_2_O_2_ production by NADPH oxidases in Arabidopsis [94]. Consistently, module M1 also included one *USP* ortholog already described [38] and one maize NADPH oxidase ortholog *ZmRbohB*. Co-expression of these genes in module M1, together with the identification of the TGACG and GCCGCC motifs (included in the maize PCO ortholog *AC203989*.*4_FG001* and the *USP* gene) and the recent observation that methyl jasmonate and SA are able to modify the cell wall structure in Brachypodium [95], thus opens the exciting possibility that ERF-VIIs might play a role in the mechanisms whereby the QTL6.05 influences cell wall degradability.

Third, genes involved in protein folding and response to heat are co-expressed in the network, including *ZmHSP40* already described [38] and three maize *HSP90* orthologs. HSP40 proteins can initiate a conserved chaperone assembly line that mediates conformational changes required for the activity of many native proteins, including HSP70 and HSP90. In plants, HSP90 chaperone pathway plays crucial role in development, and known clients include auxin and JA receptors [96,97]. Furthermore, transcripts of the Arabidopsis *HSP90* orthologs of *GRMZM2G024668* and *GRMZM2G069651* were reported to move in root-to-shoot direction [98], which may indicate that they function in shoot. Overall, these results also implicate protein folding in the mechanisms underlying the QTL6.05 effects.

### Identification of four candidate genes for the targeted QTL6.05 locus

Finally, combining meta-analysis of gene co-expression network, phenotypic NIL data and specific TF binding sites within promoters revealed *ZmFKB20-1*, *ZmESMD1*, *ZmAKR4C9* and *ZmRPS23* as promising candidate genes for the targeted QTL6.05 locus. The first two genes belonged to module M4. *ZmFKB20-1* which was one of only two DE genes already identified in the targeted QTL6.05 support interval [38] belongs to the FK506-binding protein family, which contains up to 30 paralogous genes in the maize genome [77,99]. Although historically linked to immunosuppression and proline bond rotation, the physiological importance of FK506-binding proteins extends general protein folding to diverse biological processes, including hormone signaling, DNA transcription and protein trafficking. In module M4, *ZmFKBP20-1* was co-expressed with a limited set of other genes, including *ZmEXO70B1* involved in cell polarity and morphogenesis [100] and *ZmESMD1* involved in cell adhesion through as yet unidentified cell wall modifications in Arabidopsis [78]. Together, these results indicate that *ZmFKBP20-1* may function in cell wall-related trait variation and highlight the potential importance of cell adhesion in the mechanisms underlying the QTL6.05 effects.

The two other candidate genes *ZmAKR4C9* and *ZmRPS23* belonged to module M1. They both had high connectivity with other DE genes in the gene co-expression network, including the two *ERF-VIIs* and eight of the nine DE genes displaying SPE_patterns, which were found to be associated with cell wall-related traits. Comparison of multiple sequence alignments suggests that ZmAKR4C9 may be involved in detoxification mechanisms similar to those of AtAKR4C9 [101]. Consistently, gene expression of tomato *AKR4C9* was shown to be induced by hydrogen peroxide and plant hormones such as SA and JA [102], which coincide with the enriched biological processes we observed here. In contrast to *ZmAKR4C9*, *ZmRPS23* belongs to the ribosomal protein S12/S23 family that functions in translation fidelity [103]. Interestingly, several studies identified RPS23 as an enzyme substrate for the oxygenase OFD1 in fission yeast [104]. When little oxygen is around, OFD1 cannot hydroxylate RPS23. This traps OFD1 away from a TF called SRE1. As a result, unassembled RPS23 regulates SRE1 signaling by sequestering OFD1 in an oxygen-dependent manner, thereby coupling hypoxic gene expression to rates of ribosomal synthesis. Therefore, our findings raise the intriguing possibility that unassembled ZmRPS23 regulates an as yet undetermined TF-mediated signaling pathway in which ERF-VIIs may be involved.

## Conclusion

We expanded maize genetic and genomic resources to shed light on the mechanisms whereby the QTL6.05 locus influences cell wall degradability. Our data showed that the F288 alleles exploit processes related to oxidation-reduction, regulation of hydrogen peroxide metabolism and protein folding to modify cell wall degradability. Involvement of the SA, JA and ethylene pathways in the mechanisms underlying the QTL6.05 effects is also supported by expression of several related genes, identification of the TGACG and GCCGCC regulatory motifs, and co-expression of two *ERF-VIIs* that function as central nodes in the gene co-expression network. Overall, our work led to the identification of a subset of potential new cell wall regulatory genes that could be exploited to fine-tune cell wall degradability.

## Supporting information

S1 Fig Allelic variation in NILs introgressed into targeted QTL6.05^i^. The two selected pairs of NILs (BC2-S2) were field grown under well-watered conditions for DNA and mRNA sampling and phenotyping. DNA samples were used for genotyping by PCR using three markers located in the introgressed genomic region (bngl1702, bnlg1732 and bnlg345) and one outside (umc1127) as indicated in the S2 Table. L: 50 bp DNA ladder.
(PDF)

S2 Fig F271-specific sequence identification carried out to build the panB reference genome. (i) Simulation of reads from the F288 genomic sequence using wgsim v.0.3.0. (i-ii) Alignment of F288 simulated reads and B73 (AGPv3) real reads to the F271 genomic sequence using Bowtie2 v2.2.3. (iii) Alignment of 2,322 representative transcript assemblies (RTAs) [34] to the F271 genomic sequence using gmap v.2011-08-15. (i-iii) Hard masking (with N) of common F271 genomic sequence. The same pipeline was followed to identify F288-specific sequences in the F288 genomic sequence. Together, the identified F271- and F288-specific sequences were added to the maize B73 reference genome (AGPv3) to which 2,322 RTAs were also added.
(PDF)

S3 Fig PLM distribution in promoters of the five DE genes located in the QTL6.05 locus. PLMs colored red and blue indicate PLMs specifically present in the promoter of the considered DE gene in F271 and F288, respectively. 2: ASF1MOTIFCAMV; 5: MYBCOREATCYCB1; 6: TATCCAOSAMY; 7: ARR1AT; 8: MYCATERD1; 9: WBOXNTCHN48; 11: SITEIIATCYTC; 12: SORLIP2AT; 13: DPBFCOREDCDC3; 14: MARTBOX; 15: TATA-box; 18: part of VOZ-binding sequence; 19: TATABOX4; 21: VIP1 response elements. For detailed explanation, see ‘Materials and Methods‘ and S8 Table.
(PDF)

S1 Table Genetic variation in the two selected BC2 30–16 and 31–04 maize lines. (XLSX)

S2 Table List of primer sequences used in the study. (XLSX)

S3 Table Genes found in B73 AGPv3, F271 and F288 within the QTL6.05 interval. (XLSX)

S4 Table Phenotypic values in the two pairs of NILs introgressed into targeted QTL6.05^i^. (XLSX)

S5 Table Comprehensive list of the 125 DE genes displaying differential expression between the NILs^F288^ and the NILs^271^. (XLSX)

S6 Table Genes differentially expressed in the NILs^F288^ relative to the NILs^F271^ with expression profiles positively or negatively correlating with at least one of the cell wall-related traits. (XLSX)

S7 Table PCCs for the 116 genes that are differentially expressed in the NILs^F288^ relative to the NILs^271^ and co-expressed based on a |PCC| > 0.995. (XLSX)

S8 Table List of PLMs identified in promoters of genes that are differentially expressed in the NILs^288^ relative to the NILs^271^. (XLSX)

S9 Table Relative expression values in the two pairs of NILs introgressed into targeted QTL6.05^i^. (XLSX)
