# Supplementary material for: A systems biology approach uncovers a gene co-expression network associated with cell wall degradability in maize
Source: PLoS One. 2019 Dec 31;14(12):e0227011. doi: 10.1371/journal.pone.0227011 (PMC6938352; doi:10.1371/journal.pone.0227011)
Supplement: S1 Fig — The two selected pairs of NILs (BC2-S2) were field grown under well-watered conditions for DNA and mRNA sampling and phenotyping. DNA samples were used for genotyping by PCR using three markers located in the introgressed genomic region (bngl1702, bnlg1732 and bnlg345) and one outside (umc1127) as indicated in the S2 Table. L: 50 bp DNA ladder. (PDF) [file pone.0227011.s001.pdf]

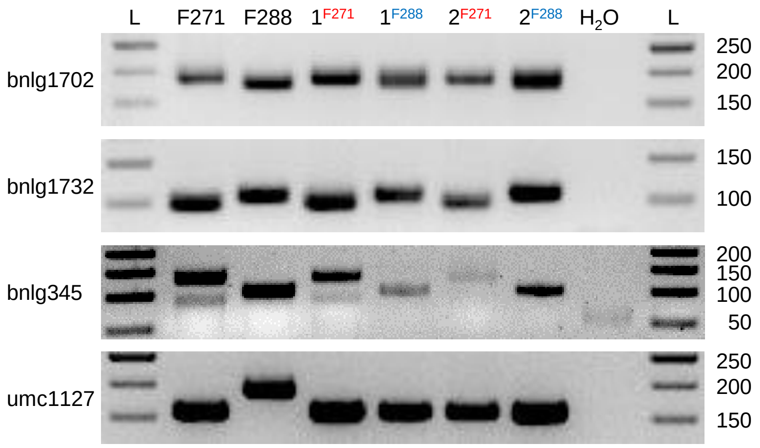

S1 Fig. Allelic variation in NILs introgressed into targeted QTL6.05[i]. The two selected pairs of NILs (BC2-S2) were field grown under well-watered conditions for DNA and mRNA sampling and phenotyping. DNA samples were used for genotyping by PCR using three markers located in the introgressed genomic region (bngl1702, bnlg1732 and bnlg345) and one outside (umc1127) as indicated in the S2 Table. L: 50 bp DNA ladder.
