# Supplementary material for: A systems biology approach uncovers a gene co-expression network associated with cell wall degradability in maize
Source: PLoS One. 2019 Dec 31;14(12):e0227011. doi: 10.1371/journal.pone.0227011 (PMC6938352; doi:10.1371/journal.pone.0227011)
Supplement: S2 Fig — (i) Simulation of reads from the F288 genomic sequence using wgsim v.0.3.0. (i-ii) Alignment of F288 simulated reads and B73 (AGPv3) real reads to the F271 genomic sequence using Bowtie2 v2.2.3. (iii) Alignment of 2,322 representative transcript assemblies (RTAs) [34] to the F271 genomic sequence using gmap v.2011-08-15. (i-iii) Hard masking (with N) of common F271 genomic sequence. The same pipeline was followed to identify F288-specific sequences in the F288 genomic sequence. Together, the identified F271- and F288-specific sequences were added to the maize B73 reference genome (AGPv3) to which 2,322 RTAs were also added. (PDF) [file pone.0227011.s002.pdf]

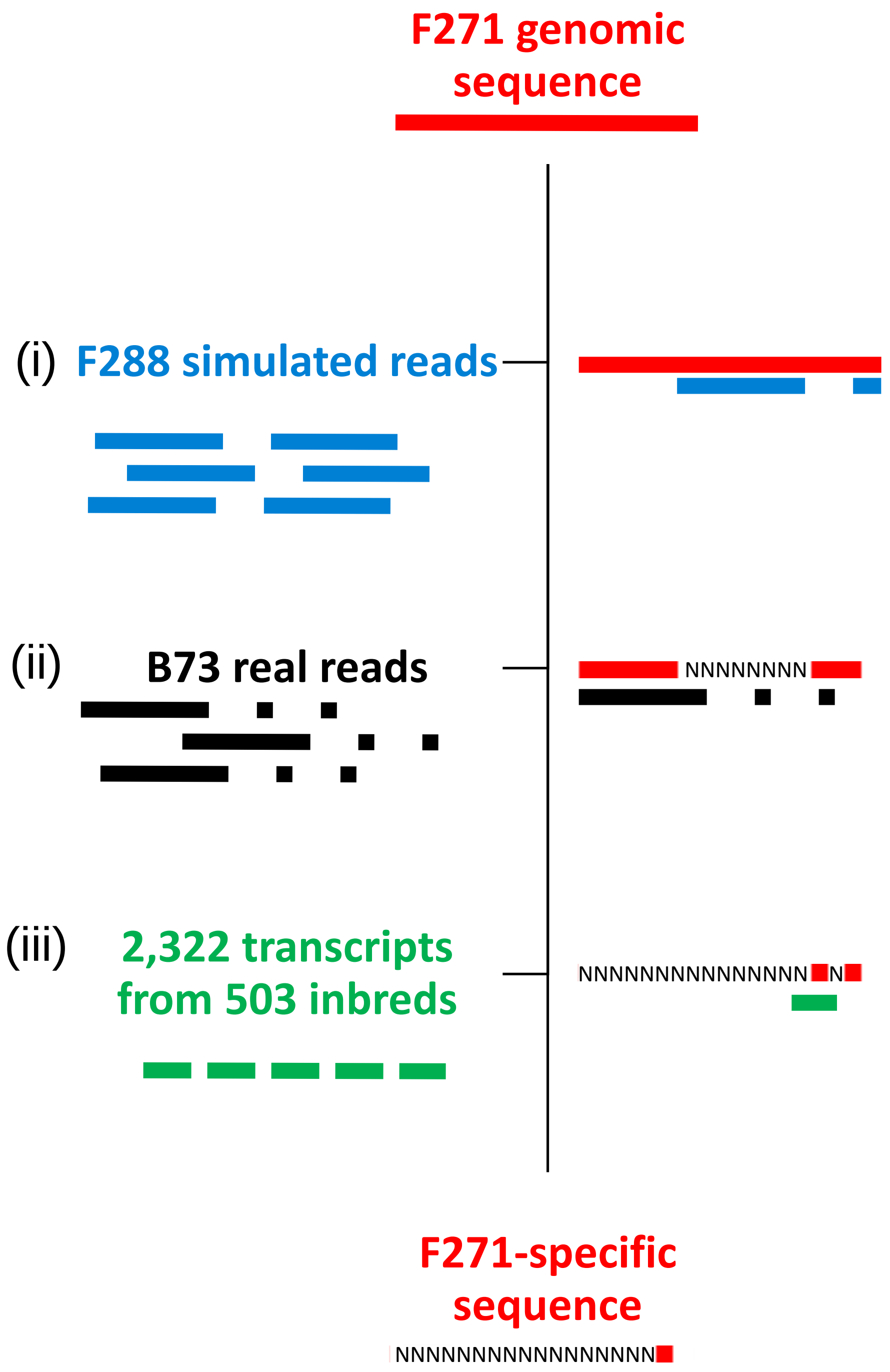

S2 Fig. F271-specific sequence identification carried out to build the panB reference genome. (i) Simulation of reads from the F288 genomic sequence using wgsim v.0.3.0. (i-ii) Alignment of F288 simulated reads and B73 (AGPv3) real reads to the F271 genomic sequence using Bowtie2 v2.2.3. (iii) Alignment of 2,322 representative transcript assemblies (RTAs) [34] to the F271 genomic sequence using gmap v.2011-08-15. (i-iii) Hard masking (with N) of common F271 genomic sequence. The same pipeline was followed to identify F288-specific sequences in the F288 genomic sequence. Together, the identified F271- and F288-specific sequences were added to the maize B73 reference genome (AGPv3) to which 2,322 RTAs were also added.
