# Supplementary material for: A systems biology approach uncovers a gene co-expression network associated with cell wall degradability in maize
Source: PLoS One. 2019 Dec 31;14(12):e0227011. doi: 10.1371/journal.pone.0227011 (PMC6938352; doi:10.1371/journal.pone.0227011)
Supplement: S3 Fig — PLMs colored red and blue indicate PLMs specifically present in the promoter of the considered DE gene in F271 and F288, respectively. 2: ASF1MOTIFCAMV; 5: MYBCOREATCYCB1; 6: TATCCAOSAMY; 7: ARR1AT; 8: MYCATERD1; 9: WBOXNTCHN48; 11: SITEIIATCYTC; 12: SORLIP2AT; 13: DPBFCOREDCDC3; 14: MARTBOX; 15: TATA-box; 18: part of VOZ-binding sequence; 19: TATABOX4; 21: VIP1 response elements. For detailed explanation, see ‘Materials and Methods‘ and S8 Table. (PDF) [file pone.0227011.s003.pdf]

ZmP5CS1_F271	-------8-----13-----18-----
ZmP5CS1_F288	-------8-----13-----18-----
ZmAKR4C9 _F271	-----------------18-----
ZmAKR4C9_F288	--------9-----------21--
ZmFKBP20-1_F271	------7----11--13-----18-----
ZmFKBP20-1_F288	-2----------12-13-----18-----
ZmESMD1_F271	-----6-7------13--15----19----
ZmESMD1_F288	----5-----------------
ZmRPS23_F271	-2---5---------14---------
ZmRPS23_F288	-2---5--7-------14---------

S3 Fig. PLM distribution in promoters of the five DE genes located in the QTL6.05 locus. PLMs colored red and blue indicate PLMs specifically present in the promoter of the considered DE gene in F271 and F288, respectively. 2: ASF1MOTIFCAMV; 5: MYBCOREATCYCB1; 6: TATCCAOSAMY; 7: ARR1AT; 8: MYCATERD1; 9: WBOXNTCHN48; 11: SITEIIATCYTC; 12: SORLIP2AT; 13: DPBFCOREDCDC3; 14: MARTBOX; 15: TATA-box; 18: part of VOZ-binding sequence; 19: TATABOX4; 21: VIP1 response elements. For detailed explanation, see ‘Materials and Methods’ and S8 Table.
